# Protecting-Group-Free and Minimally Protected Glycosylation: Recent Advances Toward Direct Glycosidic Bond Formation

**DOI:** 10.3390/molecules31203733

**Published:** 2026-10-17

**Authors:** Zhenfei Zhou, Zhongmin Yang

**Affiliations:** School of Pharmacy, Taizhou University, Taizhou 225300, China; zhouzhenfei0211@outlook.com

**Keywords:** glycosylation, protecting-group-free, native sugars, glycosyl radicals, sulfonyl hydrazides, redox-neutral cross-coupling, step economy, stereoretentive coupling, C-glycosides, nickel catalysis

## Abstract

Protecting-group-free glycosylation reduces the steps needed to convert native sugars into glycosides, but selectivity and product isolation remain major limits. This review examines methods published mainly from 2024 to August 2026. It compares donor preparation, activation conditions, linkage scope and stereochemical control. Glycosyl sulfonyl hydrazides can be prepared directly from reducing sugars and used in nickel-mediated arylation or copper-mediated C-, S- and Se-glycosylation. These donors can be isolated in large quantities by crystallization, but coupling often requires chromatography and high catalyst or reagent loadings. Ligand choice can give access to both anomers, although some methods require temporary hydroxyl protection. Photochemical thioglycoside activation, aqueous S-glycosylation and direct anomeric activation provide routes to other products. Selected examples are compared by reaction scale, isolated yield, purification and protection requirements. The comparison shows how simpler donor preparation can shorten a synthesis and why selectivity, reagent use and product isolation still affect method choice.

## 1. Introduction

Carbohydrates take part in molecular recognition, cell adhesion, immune responses and protein transport [1,2,3]. In C-glycosides, carbon replaces the glycosidic oxygen. Many C-glycosides resist enzymatic and acid-catalyzed cleavage better than the corresponding O-glycosides, which makes them useful in medicinal chemistry. SGLT2 inhibitors are established examples [4,5,6,7]. S-, Se- and N-glycosides also provide sugar analogues and conjugation products, but their stability depends on their structure and biological setting [8]. C-mannosylation can also affect protein folding and stability [9].

Classical glycosylation often uses hydroxyl protection and donor activation to control reactivity and stereoselectivity [10,11,12]. These steps can make up a large part of a synthesis. Native reducing sugars have several hydroxyl groups with similar reactivity. Their direct use therefore requires selective activation and bond formation with few or no protection steps [13,14].

Selective anomeric activation provides a basis for this approach (Figure 1). DMC (2-chloro-1,3-dimethylimidazolinium chloride) and related dehydrating reagents allow reducing sugars to be converted directly into oxazolines, azides and thioglycosides in water [15,16,17,18]. These reactions use the distinct reactivity of the anomeric position. Radical methods extend this chemistry to C–C bond formation and other coupling reactions.

### 1.1. Donors Prepared Directly from Native Sugars

Jiang, Koh, Davis and co-workers prepared a 2,3,5,6-tetrafluoropyridin-4-yl (TFP) thioglycoside from a native sugar in one step and used it for radical coupling. They called this sequence “cap-and-glycosylate” [19]. The anomeric group allows radical formation while the hydroxyl groups remain free. Tolerance of water and complex partners depends on the full reaction conditions, including the catalyst, additives and radical traps.

An isolated donor allows preparation and activation to be studied separately [20,21]. A useful comparison therefore includes donor yield and isolation as well as coupling conditions. Crystallization can make a donor easier to prepare in quantity, even when the final product requires chromatography.

Sulfonyl hydrazides provide a recent example. Earlier work established redox-neutral radical coupling [22,23] and glycosyl hydrazide preparation [24,25,26]. Gao, Hu and co-workers then used unprotected glycosyl sulfonyl hydrazides for thermal C-arylation with aryl iodides [27]. Hong, Chi and co-workers reported copper-mediated coupling [28], and Guo and co-workers developed several glycohydrazide reactions [29]. Lu and co-workers used tailored tridentate ligands to couple aryl bromides [30]. Wang and co-workers obtained either anomer by changing the ligand and conditions, but their route requires C4,C6-silylation followed by desilylation [31]. Its unprotected final products therefore do not establish a fully protecting-group-free route. A related denitrogenative study is cited as a preprint [32].

### 1.2. Scope, Methodology and Relationship to Existing Reviews

Several reviews cover closely related chemistry. Niu [20], Koh [21], Mandal [33] and Du [34] discuss radical glycosylation by donor type, while Mukherjee reviews visible-light reactions of glycosyl halides [35]. Wang, Zhu and Yang focus on radical glycosylation of fully unprotected sugars [36]. Their review covers dithiocarbamates, sulfoxides, allyl sulfones, sulfinates, polyfluorinated thiopyridines and O-based precursors. Loh discusses catalyst-controlled stereoselectivity, mainly with protected substrates [37]. Fischer and Pedersen review glycosylation in water, including DMC chemistry [38]. Dey and Jayaraman cover direct anomeric reactions of unprotected carbohydrates, with an emphasis on ionic and base-mediated methods [39].

Other reviews cover reactions that do not form glycosidic bonds. Mondal discusses isomerization, Knoevenagel reactions, selective acylation and conversion of sugars into useful chemicals [40]. Kärkäs reviews site-selective C–H reactions [41]. Taylor reviews selective hydroxyl functionalization [13] and carbohydrate photochemistry [42]. Table 1 compares these reviews with the present account.

This review compares donor preparation and coupling as parts of the full route from sugar to product. Earlier reviews cover unprotected radical donors [36] and direct anomeric reactions [39]. Li and co-workers organize visible-light glycosylation by activation mechanism [43]. Here, the comparison includes photochemical, thermal and ionic methods, with attention to temporary protection, purification and stereochemical control.The table at the back gives selected experimental examples, and Section 7.5 explains how these data can guide method choice. The Li review was published after the primary-literature cutoff and is included for context.

The main scope is glycosidic bond formation from native sugars or donors made with few preparative steps. Protecting-group-free means that non-anomeric hydroxyl groups are not deliberately masked and later unmasked. An anomeric activating group is treated as part of the donor, rather than as a hydroxyl protecting group. Minimally protected means that a route uses a limited, stated protection pattern. Transient activation means that a reactive anomeric species is formed in situ without donor isolation; it does not define the hydroxyl protection state. In situ silylation of non-anomeric hydroxyls is still a protection step. Each comparison distinguishes the starting sugar, donor, coupling substrate and final product. Reactions at C2–C6 are discussed separately in Section 6.6 because they do not form an anomeric glycosidic bond.

This article is a selective narrative review of the primary literature published mainly in 2024–2026 through 31 August 2026. Earlier studies are included where needed to explain donor preparation or mechanism. During revision on 2–5 October 2026, targeted searches used article titles, DOIs and combinations of “unprotected sugar”, “native sugar”, “protecting-group-free”, “glycosyl radical”, “sulfonyl hydrazide” and “electrochemical glycosylation”. Publisher records, accessible articles and supporting information were checked for relevant examples. Selection was based on donor preparation, protection state, activation conditions, linkage scope and stereocontrol. The Lu study [30] falls within the cutoff; the later review [43] is included for comparison with related reviews. Journal versions of articles are cited where identified, and ref. [32] remains designated as a preprint. Statements about gaps refer to the literature considered here.

### 1.3. Recent Advances and Remaining Constraints

Recent methods expand the use of donors made from native sugars. Glycosyl sulfonyl hydrazides allow thermal coupling with aryl iodides [27,29], ligand-dependent coupling with aryl bromides [30], and copper-mediated formation of C-, S- and Se-glycosides [28]. Temporary hydroxyl protection and ligand choice can give access to both anomers [31]. Aqueous radical glycosylation also allows sugar attachment to disulfide-containing biomolecules [44]. These methods address different needs and should be compared using the full synthetic sequence.

Donor-preparation burden has been reduced for several common pyranohexoses. For example, crystalline glycosyl sulfonyl hydrazides can be prepared on a decagram scale [29,30]. Their coupling products, however, may require chromatographic isolation. Catalyst loading, donor excess and substrate-dependent selectivity therefore remain relevant when comparing the full synthesis.

Earlier donor chemistry provides the basis for comparison with recent primary studies. We compare preparation, activation, linkage scope and selected routes.

## 2. Conceptual Framework

### 2.1. A Four-Part Benchmark

Yield and anomeric ratio describe the coupling step. A comparison starting from a native sugar must also include donor preparation and isolation. We use four criteria: donor-preparation burden, activation compatibility, linkage diversity and source of stereochemical control (Figure 2).

Donor-preparation burden records the chemical transformations, isolated intermediates and purification steps between the native sugar and the coupling substrate. Telescoped protection and deprotection still count as transformations. Glycosyl sulfonyl hydrazides can be isolated by precipitation or recrystallization [27,29,30], whereas DHP esters require Mitsunobu esterification [45]. These differences describe preparative work; monetary or environmental costs require additional reagent, solvent and process data.

Activation compatibility describes the tolerance of free hydroxyls, water and other functional groups under the full reaction conditions. Radical formation can be selective, but oxidants, metal reductants, bases, catalysts and solvents may limit the use of sensitive substrates. A redox-neutral reaction is not necessarily more compatible.

Linkage diversity records the anomeric C-, O-, N-, S- or Se-linked products demonstrated for a donor architecture under specified conditions. The copper-mediated sulfonyl hydrazide reactions broaden this scope [28]. Transfer to non-anomeric positions is a separate property and is excluded from the glycosidic-linkage count (Section 6.6).

The source of stereochemical control may be the substrate, catalyst, ligand or a stereoretentive reaction step. More than one factor can contribute. A product ratio alone does not explain selectivity. Comparisons must state the sugar, donor protection state, conditions and evidence for the stereochemical assignment.

### 2.2. Why the Anomeric Hydroxyl Can Be Capped Selectively

The anomeric position has distinct reactivity because of its hemiacetal structure and ring–chain equilibrium. These features allow selective reactions under dehydrative or basic conditions [16,38,39]. A numerical comparison of hydroxyl acidity would require measurements on defined compounds under stated conditions. Such values are not needed to explain the carbonyl-selective condensation below.

Sulfonyl hydrazides react with the open-chain carbonyl of a reducing sugar, followed by formation of the cyclic glycosyl derivative [24,25,26]. This gives a preferential reaction at the reducing end under the reported conditions. Petasis chemistry also uses the open-chain form, through a different reaction sequence [46].

Ring opening and closure do not guarantee retention of the starting anomeric configuration. Product stereochemistry must therefore be established from the individual transformation. Depending on the method, it may reflect radical capture, metal coordination, catalyst or ligand effects, or a stereoretentive pathway.

### 2.3. Glycosyl Radicals: What Is and Is Not Controllable

Axial/equatorial, α/β and 1,2-cis/1,2-trans describe different relationships. Axial and equatorial refer to ring conformation, while α and β describe anomeric configuration. Cis and trans compare the anomeric substituent with the C2 substituent. In the usual ^4^C_1_ chair of D-gluco- and D-galactopyranosides, axial α is 1,2-cis and equatorial β is 1,2-trans. For D-mannopyranosides in the same chair, axial α is 1,2-trans and equatorial β is 1,2-cis. Other sugar configurations and ring forms require separate assignments. A preferred radical face therefore does not imply a universal 1,2-trans product [47,48].

Stereoselectivity depends on the method and sugar. The thermal nickel studies give selective C-arylation but do not establish a general rule for radical attack [27,29]. In Lu’s aryl bromide coupling, tridentate ligands affect reactivity and anomeric selectivity [30]. Wang’s method gives α- or β-products by changing the ligand and conditions, using minimally protected precursors followed by desilylation [31]. It includes β-mannosides, which are 1,2-cis. Radical-trapping tests support radical involvement, but the origin of mannose selectivity remains unresolved. Stereoretentive glycohydrazide coupling is a separate pathway and must be assessed using the configuration and protection state of the specific precursor [23,29]. Calculated intermediate energies or comparisons with other metal systems [49,50] do not by themselves establish product selectivity.

### 2.4. Donor Types and Preparation

Donors can be grouped according to their preparation at the anomeric position. Condensation gives glycosyl sulfonyl hydrazides; substitution gives thioglycosides and pyridinium salts; and esterification gives donors such as DHP esters [19,24,25,26,45,51]. Other methods generate the reactive species in situ. These avoid isolation of a donor, but the activating reagents and any protection steps remain part of the synthesis.

## 3. Donor Preparation and Direct Activation

### 3.1. Glycosyl Sulfonyl Hydrazides

Glycosyl sulfonyl hydrazides are made by condensing reducing sugars with arenesulfonyl hydrazides. Guo and co-workers isolated 33.1 g of glucosyl tosylhydrazide in 95% yield by precipitation and filtration [29]. Lu and co-workers obtained 27.3 g of the 3,5-difluorobenzenesulfonyl derivative in 74% yield after recrystallization [30]. Gao and co-workers also describe a two-step route without chromatography [27]. Earlier uses include sugar characterization [24], O-glycosylation [25,52], glycosyl 1-phosphate synthesis [26] and glycan immobilization [53].

The sulfonyl hydrazide group can be installed selectively at the reducing end and can take part in electron transfer during coupling [22,27,29]. Nitrogen loss helps generate the radical. The sulfonyl group also affects reactivity, and different methods use tosyl or fluorinated arylsulfonyl groups. Comparisons should include their preparation and the removal of sulfur-containing by-products.

Applications include thermal nickel coupling with aryl iodides [27,29] or aryl bromides [30], and copper-mediated coupling [28]. Wang’s method gives access to either anomer [31]. Fully unprotected donors gave poor conversion, and effective coupling required a C4,C6-silylated precursor, isolated or formed in situ, followed by desilylation. Figure 3 and Tables 2, 4 and 5 retain this distinction. The denitrogenative report is discussed separately as a preprint [32].

We compares named examples by donor and product isolation, reaction scale, catalyst loading and stereoselectivity in Table 2. Optimization yields, scope yields and preparative results are kept separate because they describe different experiments.

### 3.2. Electron-Poor Aryl Thioglycosides

TFP thioglycosides can be prepared from native sugars in one step and activated by light [19]. Related sulfur groups also support C-glycosylation, but they are not interchangeable. Zhou, Koh and co-workers used a 2,3,5,6-tetrafluorobenzonitrile-4-thioglycoside as the main donor for coupling with N-heteroarenes [54]. Cao and co-workers used a tetrafluoropyridinylthio group for reactions at different sugar positions [55]. Li and co-workers used TFP thioglycosides for C-ketonylation, including an unprotected donor example [56]. Figure 4 summarizes these reactions. Non-anomeric reactions are discussed in Section 6.6.

The sulfur group, sugar protection state and coupling partner affect the required conditions. Results obtained with one thioglycoside should not be assigned to all donors in this group.

DMC-derived glycosyl dithiocarbamates and dithioimidocarbonates provide other radical precursors [57,58]. The activation conditions differ: the dithiocarbamate route uses Bu3SnH/AIBN, whereas the dithioimidocarbonate study uses photochemical coupling. Preparation and protection state must be checked for each precursor.

### 3.3. Glycosyl Sulfinates and Sulfones

Unprotected glycosyl sulfinates can undergo coupling with free hydroxyl groups, but donor preparation must be counted from the stated starting material [44,59]. The three- to four-step preparations discussed here are not one-step activations of native sugars. An unprotected coupling substrate does not establish that its preparation was protecting-group-free.

Earlier sulfur-based radical donors are reviewed elsewhere [20,33,36]. Representative developments include glycosyl sulfoxides [60], photoredox nickel coupling of allyl glycosyl sulfones [61], and sulfinate activation for Giese addition or aryl coupling [59,62,63]. Sulfone cross-coupling can use visible-light activation [64] or other catalytic conditions [65]. Photoexcitable unprotected glycosyl borates provide a separate donor class [66], while designed glycosyl sulfides enable ketonyl C-glycoside synthesis [67]. Base-promoted O-glycosylation [68] is an ionic comparison, not a photoredox sulfinate reaction.

DNA-compatible S-glycosylation illustrates two distinct pathways [69]. In the first, DMC promotes direct reaction of an unprotected sugar with a DNA-linked thiol. In the second, a preformed allyl glycosyl sulfone undergoes photoinduced radical activation for conjugation to a DNA-linked sulfur-containing partner. Scheme 1 separates these branches. Sulfinates also support aqueous S-glycosylation, as discussed in Section 5.3.

### 3.4. Dihydropyridine Esters and Other Redox-Active Esters

Fairbanks and co-workers prepared glycosyl dihydropyridine (DHP) esters directly from unprotected sugars and used light to form C-glycosides [45]. Mitsunobu esterification with 3 equivalents each of triphenylphosphine and diisopropyl azodicarboxylate and 1.2 equivalents of triethylamine gave the glucose donor in 65% yield. Increasing the base to 3 equivalents lowered the yield to 20%. Coupling with 4CzIPN in dry acetonitrile at 85 °C gave α-C-glycosides from the tested glucose, mannose and galactose donors. The 2-deoxyribose example was tentatively assigned as β, so α-selectivity should not be treated as universal. Free sugars, 1-deoxy sugars and aza-Michael adducts were observed as by-products. Excess alkene lowered the yield, and ester migration occurred without an acceptor. Styrene gave no product, but maltose and lactose donors gave C-glycosides. Scheme 2 summarizes donor formation and coupling.

Ho, Chi and co-workers also used DHP-derived glycosyl esters prepared by a selective Mitsunobu reaction [70]. Their glucose donor was isolated in 60% yield, and coupling in DMF/H_2_O (9:1) at 86 °C gave the model product in 56% yield. Unlike the dry-acetonitrile conditions above, this method uses water as a co-solvent. Their photoredox method gives unprotected C-glycosides, including C-glycosyl amino acid derivatives. Han, Zhu, Yang and co-workers instead used glycosyl benzoates and adjusted the photocatalyst and formate additives to obtain alkyl or alkenyl C-glycosides [71]. Their unprotected mannose example gave the C-glycosylation product in 36% yield with α-selectivity. This result shows that a simple benzoate can act as a radical precursor under suitable reducing conditions. Earlier work established anomeric C–O cleavage in protected glycosyl esters [72], its use for C-glycosyl amino acids and peptides [73], and nickel/photoredox coupling of related sugar precursors [74].

DHP esters provide short routes to C-alkyl glycosides, including disaccharide examples. Their value must be weighed against the Mitsunobu reagents, reaction temperature, side reactions and product isolation.

### 3.5. DMC-Derived Donors: Scope and Instructive Limits

DMC and related imidazolinium reagents allow several anomeric derivatives to be prepared in water [18,75,76,77,78]. Their use as ionic donors does not show that they will generate useful radicals. Reviews of aqueous glycosylation provide the wider context [38,39]. The pyridinium study below shows why donor formation and radical coupling must be tested separately.

Chong, Brooksby and Fairbanks prepared glycosyl pyridinium salts with DMC or CDMBI and studied their electrochemistry and photoredox reactions [51]. They report Ered values from −1.40 to −0.93 V versus a saturated calomel electrode (SCE), with all four reductions described as irreversible. These values are not reversible half-wave or formal potentials. The measurements used a glassy-carbon working electrode, aqueous 0.1 M Na_2_SO_4_, room temperature and a scan rate of 100 mV s^−1^. Other scan rates and a separate KCl-electrolyte experiment were also tested. Despite reduction of the salts, the tested photoredox conditions did not give the intended radical-trapping products. Ortho-substitution hindered salt formation, and salt stability depended on pyridine substitution (Figure 5).

### 3.6. Transient Activation and the Donor-Free Limit

Transient anomeric activation can avoid isolation of a discrete donor, although activating reagents and reductants remain necessary. Xie, Wang and Shu used low-valent titanium generated with zinc for C–OH activation of native saccharides and radical addition to alkenes [79]. The method includes mono- and oligosaccharide examples and useful stereoselectivity. Its directness should be balanced against stoichiometric metal-reductant requirements.

Phosphorus-assisted activation provides another route. Hu and co-workers use phosphitylated 1-hydroxycarbohydrates for C-alkyl glycoside synthesis [80]. Chi and co-workers use phosphorus-assisted C–OH activation for direct arylation [81]. These methods should be assessed separately for precursor preparation and protection state. Benzodithiolane-mediated C–O activation provides a further approach [82].

In situ bromination provides a useful comparison. Zhang, Yang and Shi form a glycosyl bromide in the reaction mixture and then use nickel-catalyzed reductive coupling with acrylates [83]. The bromide is not isolated, but the starting sugars are partially protected and have only the anomeric hydroxyl free. The method therefore does not establish direct coupling of native sugars. A related visible-light-assisted coupling uses D-glucose and aryl bromides [84]. These examples show why a free anomeric hydroxyl must be distinguished from fully unprotected hydroxyl groups.

A non-radical route also avoids a permanent anomeric activating group. He and co-workers reported an “open-and-close” synthesis of 1,2-cis C-vinyl furanosides from unprotected aldoses [46]. Morpholine promotes ring opening and a Petasis reaction with a vinylboronic acid. Difluorocarbene then activates the C–N bond, allowing the C4 hydroxyl to close the furanose ring with inversion. The glucose example gave 93% yield and an 11:1 anomeric ratio. The study includes pentoses, hexoses, disaccharides and a gram-scale reaction in aqueous acetonitrile. A related halogenation-induced deaminative cyclization gives 1,2-cis C-aryl furanosides [85].

**Table 2:** Comparison of donor architectures and transient activation strategies, emphasizing accessibility from native sugars and including selected multistep donor preparations as benchmarks.

Donor Architecture	Preparation and Protection State	Purification	Bench Stability	Principal Activation	Anomeric Products	Key Refs
Sulfonyl hydrazide (UP coupling)	Native sugar → UP donor → UP coupling → UP product; one donor-forming operation	Precipitation or recrystallization [29,30]; coupling product may require chromatography	Crystalline; substrate-dependent	Thermal Ni; Ir/Ni photochemical; Cu-mediated	C-aryl/heteroaryl, C-alkenyl/alkyl, O, S, Se	[25,27,28,29,30]
Electron-poor aryl thioglycoside	One step from native sugar	See cited substrate-specific procedures	Isolable; storage depends on donor	Photoinduced desulfurative homolysis	C-alkyl, C-ketonyl and C-heteroaryl; donor-specific	[19,54,55,56]
Sulfinate	Multistep preparation; UP coupling donor; upstream route assessed separately	See cited substrate-specific procedures	Storage depends on donor and conditions	Oxidative SET (tBuOOH); photoredox	C-alkyl, C-aryl, S (aqueous)	[44,59,62,63]
Dihydropyridine ester	One step, Mitsunobu	Chromatography; anomeric mixture	Isolable; storage depends on donor	Photoredox; 4CzIPN, 85–86 °C; solvent differs	C-alkyl (Giese)	[45,70]
Pyridinium salt	One step, aqueous DMC/CDMBI	Ion-exchange chromatography/lyophilization	Substituent-dependent; some salts unstable	No intended Giese products under tested conditions	None (negative result)	[51]
Dithiocarbamate/dithioimidocarbonate	DMC-derived; preparation and protection depend on donor	See cited substrate-specific procedures	Isolable; storage depends on donor	Radical activation; thermal or photochemical	C-alkyl, C-aryl	[57,58]
Transient Ti alkoxide	In situ activation; no isolated donor	Not isolated	n/a	Low-valent Ti/Zn	C-alkyl	[79]
Phosphorus-assisted activation	Phosphorus-derived precursor; preparation and protection depend on protocol	Check individual precursor procedure	Protocol-dependent	Photoredox or Ni/Zn, depending on protocol	C-alkyl, C-aryl, N	[80,81,86]
Transient bromide	In situ bromination of partially protected sugars; bromide not isolated	Not isolated	n/a	Ni/Zn reductive coupling	C-alkyl	[83]
Morpholine (Petasis, non-radical)	Ring opening/Petasis addition, then ring closure; native-sugar route	See the cited substrate-specific procedures	Isolable; storage depends on donor	Difluorocarbene-induced deamination	1,2-cis C-vinyl and C-aryl furanosides	[46,85]
Benzoate ester	One step, selective anomeric benzoylation	See cited substrate-specific procedures	Isolable; storage depends on donor	Photocatalytic, switchable	C-alkyl and alkenyl	[71]
Sulfonyl hydrazide (MP coupling)	Native sugar → hydrazide → C4,C6-silylation → coupling → desilylation	Isolated or telescoped; final purification required	Isolable donor	Ni/ligand; 60 °C	α or β C-(hetero)aryl; UP products	[31]

Donor-preparation burden includes chemical operations as well as isolated intermediates. UP denotes unprotected non-anomeric hydroxyls; MP denotes an explicitly protected coupling-stage precursor. Transient activation does not imply zero operations. The Wang sequence [31] is classified separately because silylation and desilylation occur even when telescoped. The table at the end of the article gives representative method-level comparisons; protection state is not inferred from the final product.

## 4. Activation Modes

Donor structure and activation conditions must be considered together. Sulfonyl hydrazides can undergo nickel- or copper-mediated reactions [27,28,29,30,31], while sulfinates can be activated by light or oxidants [44,62]. Compatibility demonstrated under one set of conditions should not be assumed for another. Table 3 summarizes the comparison of activation modes.

### 4.1. Photoredox and Metallaphotoredox Catalysis

Photoredox and metallaphotoredox methods are widely used in glycosylation [8,42,87]. Examples include allyl glycosyl sulfone coupling [61], sulfinate addition and arylation [62,63], ester activation [45,70], and coupling of unprotected sugar-derived donors with N-heteroarenes [54]. Direct excitation of glycosyl borates avoids an added photocatalyst [66]. Excited-state palladium coupling of glycosyl bromides with quinoxalinones uses protected donors [88]. Other protected-carbohydrate photochemical reactions provide mechanistic context [89,90].

Photochemical scale-up depends on wavelength, light penetration, concentration and reactor geometry. Thermal reactions remove the light-delivery requirement, although heating, mixing and product isolation still require evaluation at the intended scale. Lu also reports an Ir/Ni photochemical variant for aryl bromide coupling [30]; its preparative conditions are compared with the thermal procedure in the final table.

### 4.2. Thermal, Redox-Neutral Nickel Catalysis

Sulfonyl hydrazide radical cross-coupling can operate without an external redox reagent because the precursor participates in electron transfer [22,23,91]. Calorimetric work on the general platform supports a nested multicycle mechanism [92]. Its application to a particular carbohydrate system requires separate mechanistic evidence.

Gao, Hu and co-workers report thermal coupling of unprotected glycosyl sulfonyl hydrazides with aryl iodides using nickel and tetramethylguanidine in DMSO [27]. The study includes several sugars and (hetero)aryl partners. Radical-trapping experiments and product formation from a preformed nickel complex support radical involvement and the reactivity of that complex. The proposed Ni(III)/Ni(I) comproportionation step remains a mechanistic interpretation rather than a consequence established by those experiments alone.

Lu and co-workers use tailored tridentate ligands to couple unprotected glycosyl sulfonyl hydrazides with aryl bromides [30]. Under thermal conditions, Ni(BF_4_)_2_·6H_2_O and ligand L21 (20 mol% each), TEMP-OH (3 equiv.), and DMF at 40 °C gave empagliflozin in 68% isolated yield on a 3.0 mmol aryl bromide scale, with β:α > 20:1. The same study also reports an Ir/Ni photochemical variant; the two conditions are distinguished in the final table. Guo and co-workers use Ni(dme)Cl_2_, ligand and TMG for aryl iodide or selected aryl bromide coupling [29]. Their preparative dapagliflozin example gave 7.2 g of product in 62% isolated yield after chromatography. Wang and co-workers use temporary hydroxyl protection to obtain ligand-dependent access to either anomer [31].

These examples show that donor isolation and product isolation can have different requirements. Guo’s glucosyl donor is obtained by filtration, but preparative dapagliflozin synthesis uses silica chromatography [29]. Lu’s donor is recrystallized, whereas empagliflozin is isolated by silica chromatography [30]. Thus, easier donor preparation shortens the sequence without necessarily removing its main product-purification step.

### 4.3. Copper-Mediated Single-Electron Activation

Hong and co-workers obtain alkenyl and alkyl C-glycosides, thioglycosides and selenoglycosides from unprotected glycosyl sulfonyl hydrazides [28]. The conditions vary with the partner. Alkenylation uses CuCl and a diamine ligand (1 equiv. each), KOH (3 equiv.) and HFIP at 90 °C; representative alkylation uses Cu(OTf)_2_ (0.5 equiv.) and NaOAc (3 equiv.) at 80 °C. A 1.77 g alkyl C-glycoside example was isolated in 81% yield. These loadings and the chromatographic work-up are relevant to preparative use. Copper metallaphotoredox N-glycosylation of 1-hydroxycarbohydrates follows a different activation strategy [86].

### 4.4. Low-Valent Titanium and Reductive Metal Systems

Low-valent titanium generated from Cp*TiCl3 and zinc activates the anomeric C–OH bond directly (Section 3.6) [79]. Nickel/zinc systems can couple transient glycosyl bromides [83]. Iron-catalyzed coupling of protected glycosyl donors provides a related comparison [50]. These reactions avoid light and electrolysis, but their use of metal reductants adds to reagent consumption and work-up.

### 4.5. Electrochemical Activation

Electrochemistry permits control of electrode potential and has enabled halogen-atom-transfer C-glycosylation [93]. Matsumoto and Nokami review broader carbohydrate electrochemistry [94], whereas the study cited in ref. [95] concerns ammoxidation of unprotected glycosides. The latter does not form an anomeric glycosidic bond. The pyridinium study provides reduction data but did not establish productive glycosyl radical coupling under the tested conditions [51]. Electrochemical activation of the one-step sulfonyl hydrazide donors discussed here was not identified in the literature considered for this review.

### 4.6. Photocatalyst-Free and Medium-Controlled Activation

Direct excitation of glycosyl borates avoids an added photocatalyst [66]. Glycosyl sulfinates can instead be activated with tert-butyl hydroperoxide in water, including for S-glycosylation of disulfides at 37 °C [44]. HFIP is used in copper-mediated alkenylation [28,96] and in some ionic glycosylations [97]. These examples illustrate how solvent and reagent choice determine compatibility with a particular substrate.

**Table 3:** Comparison of activation modes for glycosylation, including methods applicable to native sugars or their unprotected derivatives and selected systems that define current scope limitations.

Mode	Energy Input	Stoichiometric Additive	Typical Temperature	Aqueous Compatible	Principal Constraint	Key Refs
Photoredox/metallaphotoredox	Visible light	Protocol-dependent redox additives; photocatalyst is catalytic	25–86 °C	Protocol-dependent; e.g., DMF/H_2_O [70]	Photon flux does not scale with volume	[30,45,55,61,64,70]
Thermal redox-neutral Ni	Heat	Base; donor supplies reducing equivalents	40–70 °C	Organic solvent in examples tabulated	Catalyst/base loading; protection state varies	[22,27,29,30,31,92]
Cu-mediated SET	Heat	Cu salts (0.5–1 equiv.); base	80–90 °C	Partially (MeCN/H_2_O)	Copper loading and product chromatography	[28]
Low-valent Ti	No light or electrolysis	Zn	Ambient to mild	No	Stoichiometric metal reductant	[79]
Ni/Zn reductive	No light or electrolysis	Zn	Ambient	No	Partially protected substrates to date	[83]
Electrochemical	Electric current	Electrolyte	Ambient	Protocol-dependent	One-step sulfonyl hydrazide combination not identified in this review	[93,94]
Oxidant-mediated in water	Thermal control (37 °C)	tBuOOH	37 °C	Yes	Excess donor and oxidant required	[44]
Direct photoexcitation of glycosyl borate	Visible light	Protocol-dependent; no added photocatalyst	Ambient	Yes	Donor design constrained	[66]

Water compatibility means demonstrated use of water as solvent or co-solvent. Energy input and stoichiometric material input are listed separately. The absence of irradiation or electrolysis does not remove the need for a chemical reductant.

## 5. Linkage Diversity from a Common Donor

### 5.1. C-Aryl and C-Heteroaryl Glycosides

Aryl C-glycosides are prominent targets, including gliflozins [6,7]. Representative routes use glycosyl sulfones [61], unprotected sulfinates [63], and glycosyl sulfonyl hydrazides coupled with aryl iodides [27,29] or aryl bromides [30]. The bromide study expands electrophile choice and illustrates ligand effects on anomeric selectivity. Photocatalytic coupling to N-heteroarenes provides C-linked products [54]. Coumarin functionalization [98] and excited-state palladium coupling of protected glycosyl bromides [88] are distinct comparisons; they should not all be classified as fully unprotected routes.

### 5.2. C-Alkyl, C-Alkenyl and C-Alkynyl Glycosides

Giese-type addition provides C-alkyl products using DHP esters [45], other glycosyl esters [70], titanium-activated native sugars [79] and phosphorus-based activation [80]. In situ bromide coupling [83] is a protected-substrate comparison. Cascade reactions can construct more elaborate products, including cyclopropane-containing C-glycosides [99]. Protection state and donor preparation must be assessed independently for each route.

Alkenyl C-glycoside synthesis requires control of both anomeric configuration and alkene geometry. The copper/sulfonyl hydrazide method gives highly selective examples, but these two ratios must be reported separately [28]. Cobalt-catalyzed hydroglycosylation of alkynes gives vinyl α-C-glycosides [100]. Photochemical methods provide stereoselective or stereodivergent C-styryl products [101] and stereospecific vinylation [102]. Nickel-catalyzed reductive hydroglycosylation has also been used for C-oligosaccharides [103]. The non-radical Petasis route gives 1,2-cis C-vinyl furanosides [46].

### 5.3. S- and Se-Glycosides, Glycopeptides and Proteins

Aqueous S-glycosylation demonstrates a different capability of radical donors. Following earlier polypeptide and protein conjugation work [104], Niu and co-workers reported bis-S-glycosylation of disulfides using glycosyl sulfinates and tert-butyl hydroperoxide in water at 37 °C [44]. The reported sequence installs glycosyl units across the disulfide. Stereochemical assignments should be reported for the individual sugar rather than converted from an axial preference into a universal cis/trans rule.

The sulfinate method was applied to peptides containing free hydroxyl, amino, imidazole and guanidine groups [44]. Examples include a Jagged-1 fragment, iRGD, oxytocin and glutathione dimers. The reported sugars include glucose, galactose, mannose, amino sugars, disaccharides and a trisaccharide. Mixed disulfides allow selective installation of one sugar, while differences in disulfide reactivity allow sequential installation of different sugars on a peptide.

These applications make use of multivalent sugar display [105,106]. In the reported in vitro comparison, a peptide carrying two GalNAc units showed liver-targeting activity comparable to the trivalent GalNAc group used in an approved siRNA conjugate [44]. A glycosylated HER2-targeting affibody was linked to a chelator, labelled with gallium-68 and used for tumour imaging in mice. Blocking experiments supported target-specific uptake. Insulin was glycosylated in moderate yield, but Herceptin was not. These results define both useful applications and current substrate limits.

Other S- and Se-glycosylation methods include β-thioglycosylation of unactivated carbohydrates [107], α-thioglycoside and selenoglycoside synthesis from sulfonyl hydrazides [28], organophotoredox thioglycosylation [108], stereoretentive selenoglycoside synthesis [109], and acid-catalyzed 1,2-cis-thioglycosylation [110]. These routes differ in donor preparation and protection state. Earlier disulfide-to-thioether conversion enabled protein modification [111]. Thioglycosidically linked cyclic peptides have also been studied as models for glycoprotein drugs [112].

### 5.4. N-Glycosides

Ding and co-workers used glycosyl sulfinates for radical-assisted N-glycosylation [113]. Their proposed pathway includes a glycosyl iodide followed by stereospecific substitution. Copper metallaphotoredox coupling of 1-hydroxycarbohydrates provides another route [86], with broader context given in ref. [87]. CO2-mediated N-glycoconjugation of amines is a separate approach [114]. Coupling to the carbon atom of an N-heteroarene remains C-glycosylation [54]. Protection state differs among these methods and must be checked for each example.

### 5.5. O-Glycosides and the Boundary with Ionic Chemistry

Ionic methods remain important for O-glycosylation of native sugars [16,38,39]. Sulfonyl hydrazides have been used for O-glycosylation [25,52] and glycan immobilization [53]. Base-promoted O-glycosylation of carboxylic acids provides another example [68]. Amino-boronic acid catalysis couples minimally protected donors and acceptors [115]. Aqueous phenolic O-glycosylation of complex peptides is also possible [116]. In each case, tolerance of an unprotected acceptor does not establish that the donor is unprotected. Table 4 lists the substrate scope for various glycosidic bond construction methods.

## 6. Applications of Accessible Donors

The following examples show how donor preparation affects synthesis. Each route must also be assessed by protection state, coupling conditions and product isolation.

### 6.1. Shorter Synthetic Routes

Recent sulfonyl hydrazide methods shorten the sugar-derived portion of routes to gliflozins and other C-glycosides [27,29,30]. Such step counts start from the sugar and do not include synthesis of the aryl partner. Empagliflozin, dapagliflozin and canagliflozin are representative targets, rather than an exhaustive list of approved SGLT2 inhibitors. Neopetrosin synthesis illustrates related heteroaryl coupling [27,29,117]. The non-radical furanoside strategy provides a complementary route-shortening example from L-xylose [46].

### 6.2. Sugar Scanning

Simple donor preparation can make it easier to replace the sugar during analogue synthesis. Gao and co-workers prepared gliflozin analogues with different sugars [27]. This reduces the need to design a separate protection sequence for each sugar, but donor yield, selectivity and purification still depend on the substrate.

### 6.3. Late-Stage Diversification

Copper-mediated coupling accommodates an unprotected phenolic group in the synthesis of an anti-inflammatory C-mannoside [28]. Titanium-mediated reactions, coumarin functionalization and cascade additions offer other routes to substituted C-glycosides [79,98,99]. The choice depends on the desired linkage and on functional groups exposed to the reaction conditions.

### 6.4. Biomolecular Conjugation

Aqueous S-glycosylation and DNA-compatible reactions show how glycosylation can be used for biomolecular conjugation [44,69]. Other methods give amide-linked C-glycosyl amino acids and peptides [118], glycosyl amides [119] and carbohydrate-containing probes [29]. These methods differ in donor preparation and protection state; their compatibility is specific to the tested substrates and conditions.

### 6.5. Scale and Process

Donor and coupling scales differ in the reported examples. Guo prepared the glucosyl donor on a 100 mmol scale in 95% yield; a separate 28.7 mmol coupling gave 7.2 g of dapagliflozin in 62% yield [29]. Lu used 3.0 mmol of aryl bromide to obtain 920 mg of empagliflozin in 68% yield [30]. Hong obtained 1.77 g of an alkyl C-glycoside in 81% yield and 1.32 g of a selenoglycoside in 73% yield [28]. These results show preparative use for the stated products. Catalyst recovery, solvent use and isolation at larger scale still need study.

### 6.6. Related Reactions at Non-Anomeric Positions

The glycohydrazide strategy also enables C–C bond formation at C2–C6 [29]. These reactions require precursors prepared for the selected position and do not simply cap the anomeric hydroxyl of an unchanged native sugar. Polyfluorinated thiopyridyl groups can also be installed at C1, C5 or C6 under different conditions [55]. These studies extend an activation method to other positions, but they do not add to the range of anomeric glycosidic linkages. Their precursor and protection requirements must be assessed separately. Related native-sugar reactions include indium-catalyzed allylation [120], site-selective C–O editing [121] and boronic acid/photoredox alkylation [122]. These reactions modify the sugar framework and are outside the anomeric linkage comparison.

## 7. Critical Benchmarking

### 7.1. Progress in Donor Preparation and Its Limits

One-step preparation of crystalline donors can reduce the work needed before coupling [19,27,28,29,30]. Its value depends on the following reaction. Table 5 compares sulfonyl hydrazide examples with stated donor preparation, coupling conditions and product data. Nickel arylation gives unprotected C-aryl products, copper-mediated methods reach other linkage classes, and temporary silylation allows access to both anomers in the Wang method. Each approach has different catalyst, reagent and purification requirements.

### 7.2. What Remains Substrate-Dependent

Coverage differs among sugar classes. In the studies considered here, 2-deoxy sugars, uronic acids, sialic acids and furanoses are less widely represented than common pyranohexoses. This does not mean that suitable methods are absent. DMC reactions have substrate-specific limits [38]. Ring-opening and deaminative cyclization provide routes to furanosides [46,85], while some oligosaccharides can be used in ester and direct-activation methods [45,79]. A result for one substrate does not establish the behaviour of a whole sugar class.

### 7.3. How Protecting-Group-Free Is Protecting-Group-Free?

Protection state is recorded at four stages: starting sugar, donor preparation, coupling and final product. In the Wang method [31], a native sugar is converted to a hydrazide, C4, C6-silylation gives the coupling substrate, and desilylation gives the final unprotected glycoside. Combining steps in one vessel can reduce isolations but does not remove the protection operations. An unprotected donor may also require several earlier steps. Step count, protection count and chromatography count must therefore be reported separately.

### 7.4. What Should Be Reported

Yield comparisons require a clear limiting-substrate basis. Lu reports empagliflozin yield relative to aryl bromide, using 1.8 equivalents of glycosyl donor [30]. Guo reports the preparative dapagliflozin yield relative to glycosyl donor, using 1.5 equivalents of aryl iodide [29]. These yields describe different material balances. Table 5 states the basis and separates isolated yields from NMR optimization yields.

A useful report should state the starting material, donor yield, protection steps, coupling scale, isolated product yield, catalyst loading, reagent amounts and purification. An overall yield is meaningful when the steps belong to the same defined route. Solvent use and waste require more complete process data than most reports provide.

### 7.5. Choosing a Method

For unprotected C-aryl glycosides, the available aryl partner and the required anomer provide useful starting points. The Gao and Guo protocols use aryl iodides [27,29], while Lu provides detailed thermal and photochemical procedures for aryl bromides [30]. Guo also reports selected aryl bromides with a different ligand [29]. Crystalline hydrazide preparation is helpful when several analogues will be made from one sugar, although the need for chromatography should be assessed for the target product.

When both anomers are needed, temporary 4,6-O-protection may be useful. Wang’s ligand-dependent sequence provides this choice [31], but silylation and desilylation remain part of the route. For C-alkyl, C-alkenyl or Se-linked products, the copper-mediated method offers different coupling reactions. Copper loading and solvent use must be considered alongside yield [28].

For disaccharide C-alkylation or aqueous conjugation, other donor classes may be more suitable. DHP ester photochemistry has been demonstrated with maltose and lactose derivatives [45], while sulfinate activation supports S-glycosylation of disulfide-containing biomolecules in water [44]. These applications address substrates and linkage types that are not represented by the arylation benchmarks. Method selection should therefore begin with the target linkage, sugar and partner, followed by comparison of the complete preparative sequence.

## 8. Outlook

Further improvements in native-sugar glycosylation will depend on control of anomeric selectivity across a wider range of sugars. Ligand choice has expanded coupling with aryl bromides [30]. It also allows access to both anomers with minimally protected donors [31]. Stereoretentive coupling offers a further approach, although its preparation and protection requirements differ [23,29]. Lower catalyst loadings and simpler product isolation would increase the preparative value of these advances. Electrochemical activation of accessible sulfonyl hydrazide donors is also worth examining.

A second priority is to extend the range of useful linkages and complex substrates. Aqueous conjugation and selective N-glycosylation remain important targets, alongside integration with enzymatic synthesis. Reporting complete sugar-to-product sequences would make future methods easier to compare and reproduce.

## 9. Conclusions

Recent methods convert native sugars into several glycoside classes with little or no hydroxyl protection. Glycosyl sulfonyl hydrazides are useful because they can be prepared directly and used in several coupling reactions. The main gains are fewer preparative steps and access to a wider range of coupling partners. Selectivity and isolation still depend on the sugar and reaction. A comparison of the full sequence, including temporary protection, donor excess and purification, gives a better guide to method choice than step count or coupling yield alone.

## Figures and Tables

**Figure 1:**
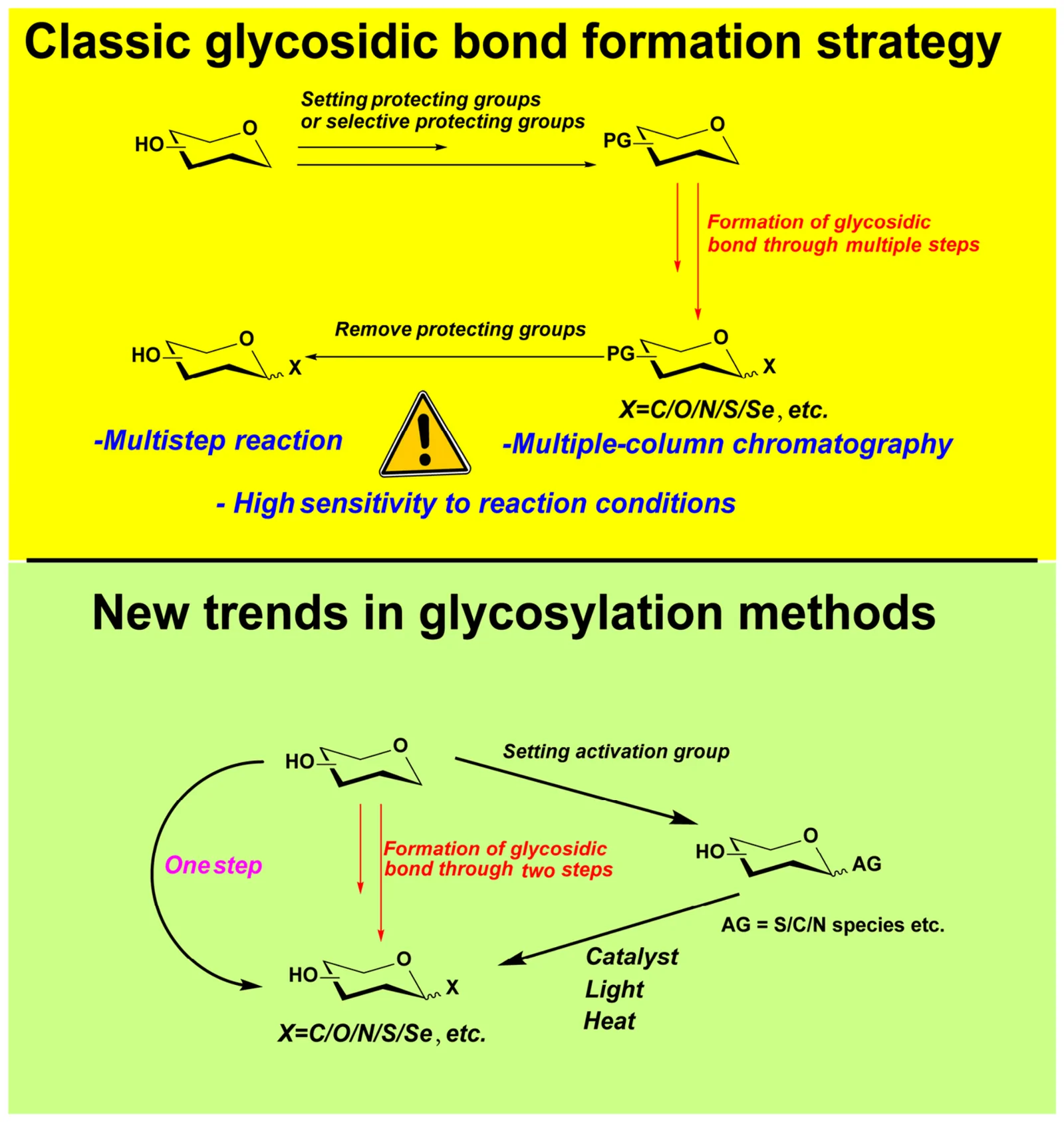
Comparison of glycosylation with hydroxyl protection and glycosylation through selective anomeric activation. The first route includes protection, bond formation and deprotection. The second uses an activating group at the anomeric position while retaining free hydroxyl groups. AG, activating group.

**Figure 2:**
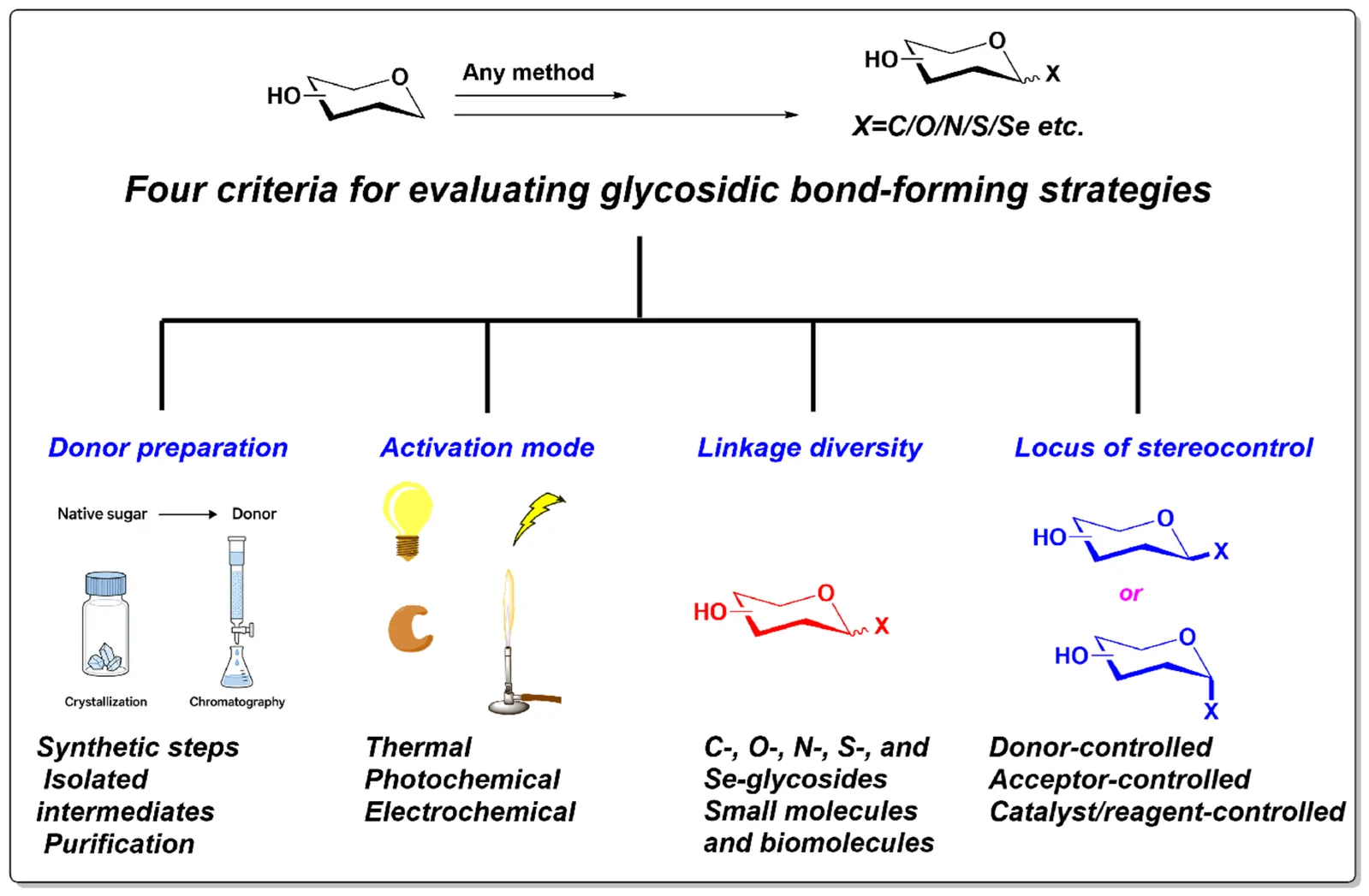
Four criteria for comparing glycosylation methods: donor preparation, compatibility of the activation conditions, anomeric linkage scope and source of stereochemical control. Donor-preparation burden includes transformations, isolated intermediates and purification; it is not a measure of monetary cost. “Locus of stereocontrol” in the diagram refers to the step or component that controls stereochemistry.

**Figure 3:**
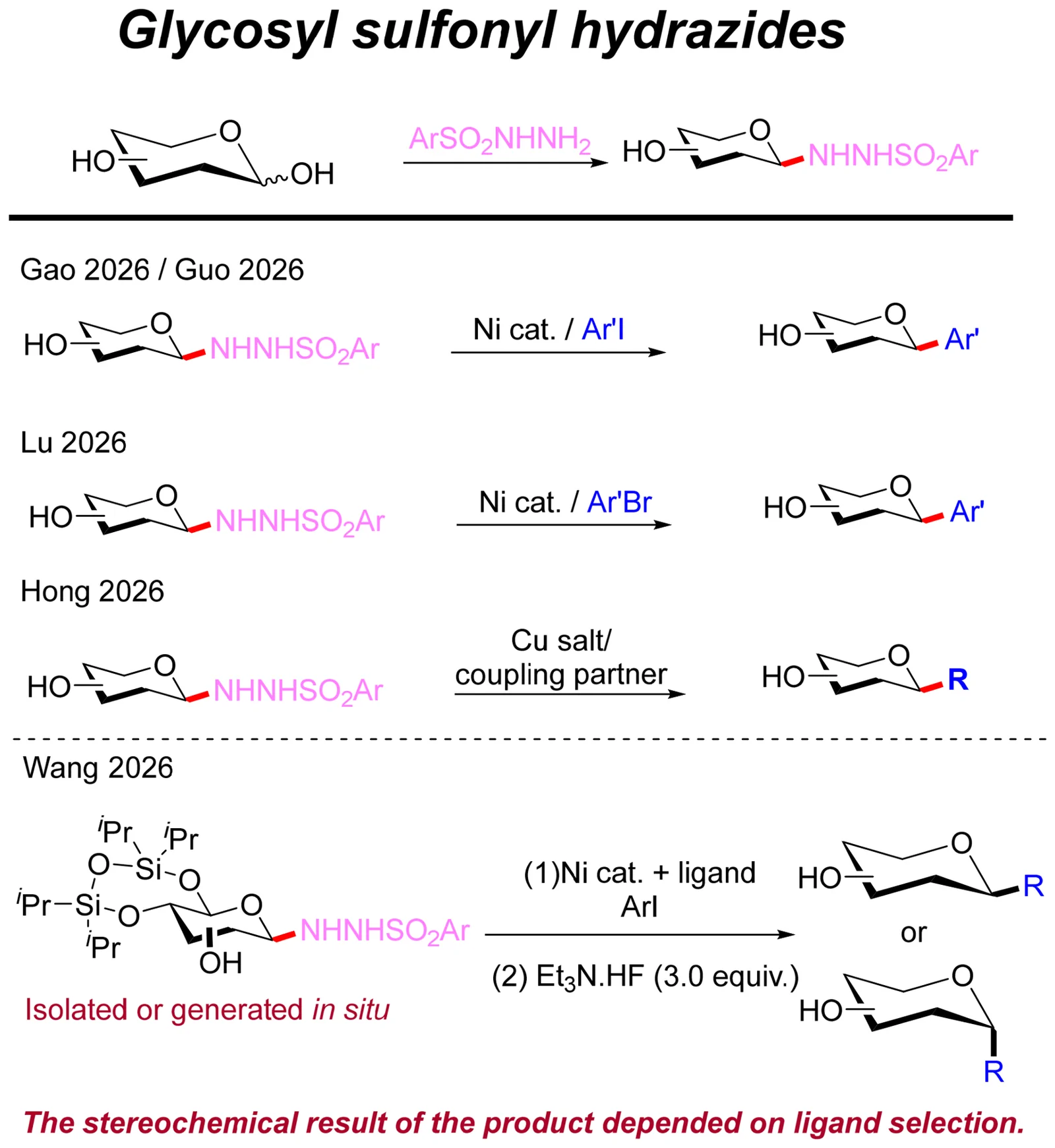
Glycosyl sulfonyl hydrazides in nickel- and copper-mediated glycosylation. The selected studies are representative examples of the methods discussed here [27,28,29,30,31].

**Figure 4:**
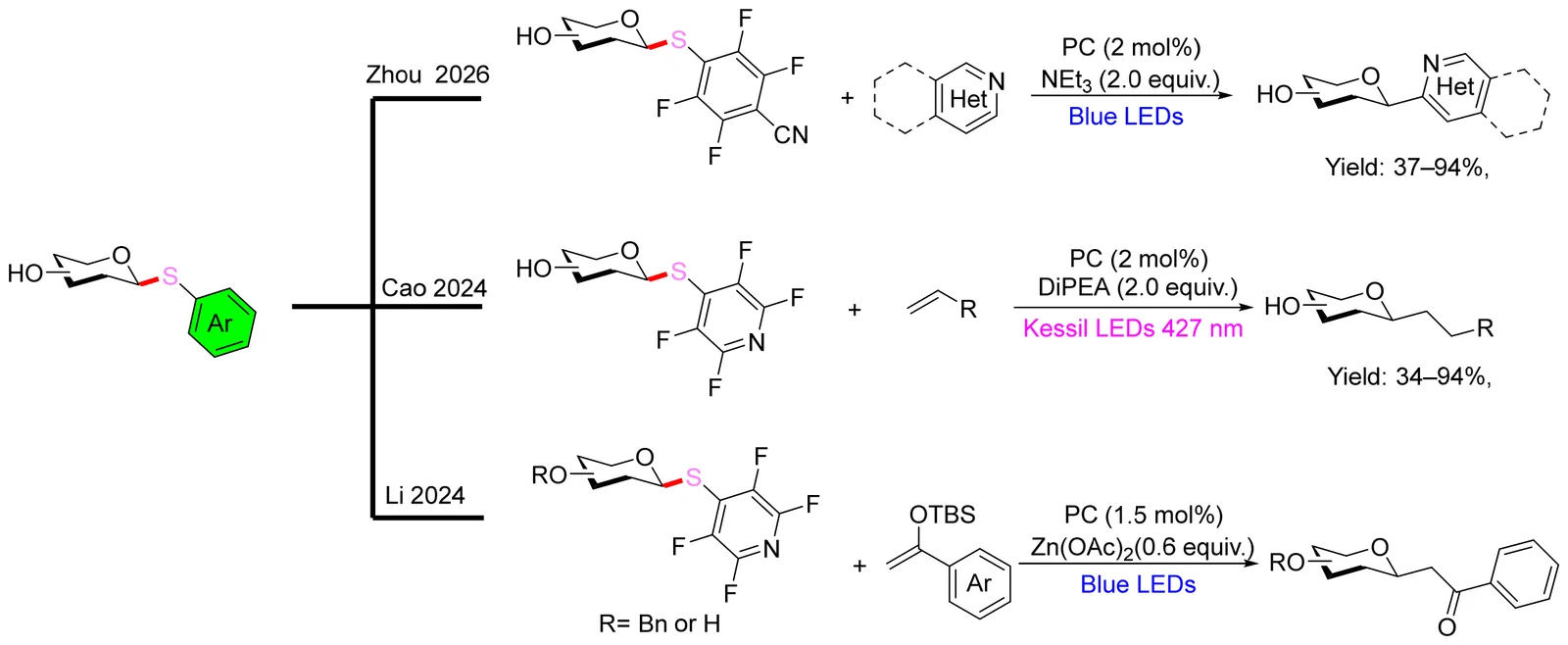
Photochemical C-glycosylation with electron-poor aryl thioglycosides. PC, photocatalyst; TBS, tert-butyldimethylsilyl; Bn, benzyl [54,55,56].

**Scheme 1:**
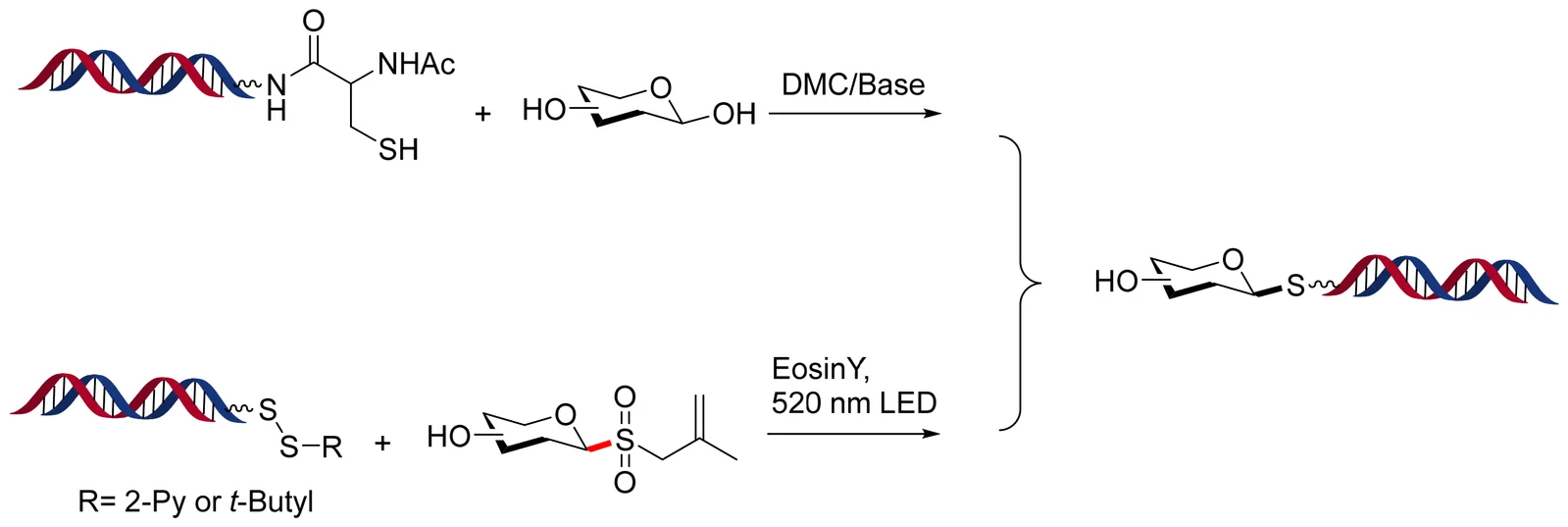
Two distinct DNA-compatible S-glycosylation pathways. The first route employs an ionic strategy, whereas the second proceeds via a radical pathway [69].

**Scheme 2:**
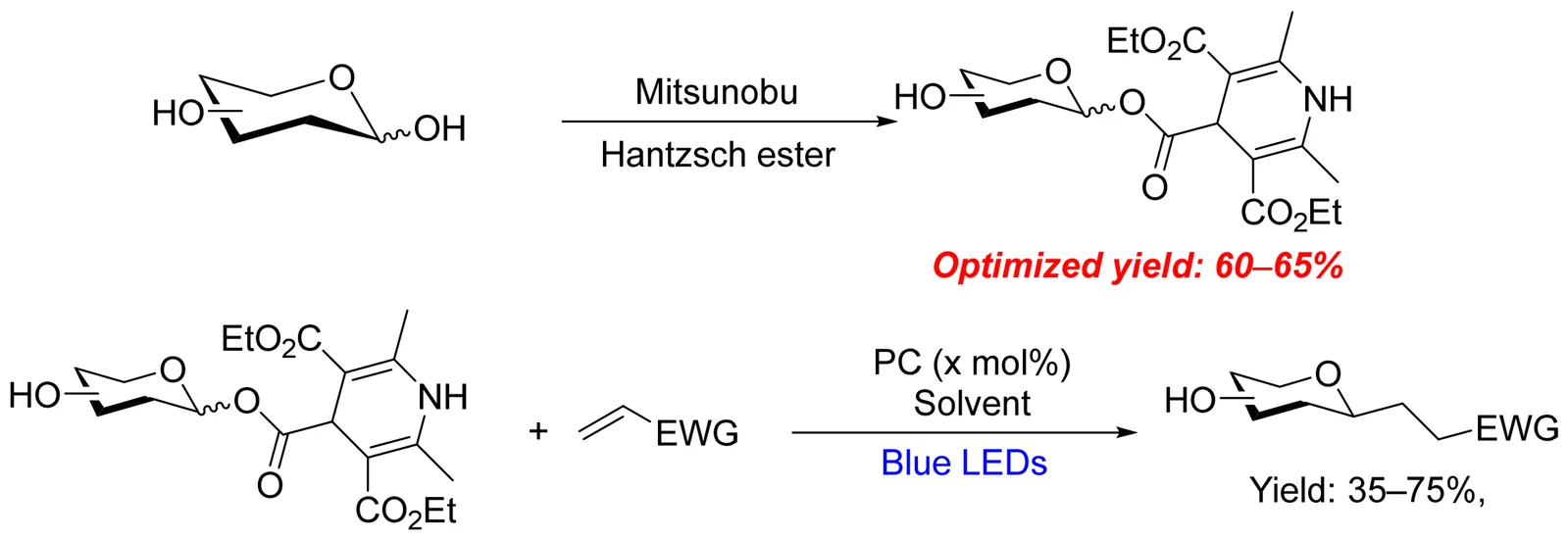
Visible-light-mediated C-alkyl glycosylation of unprotected sugars via glycosyl Hantzsch ester intermediates.

**Figure 5:**
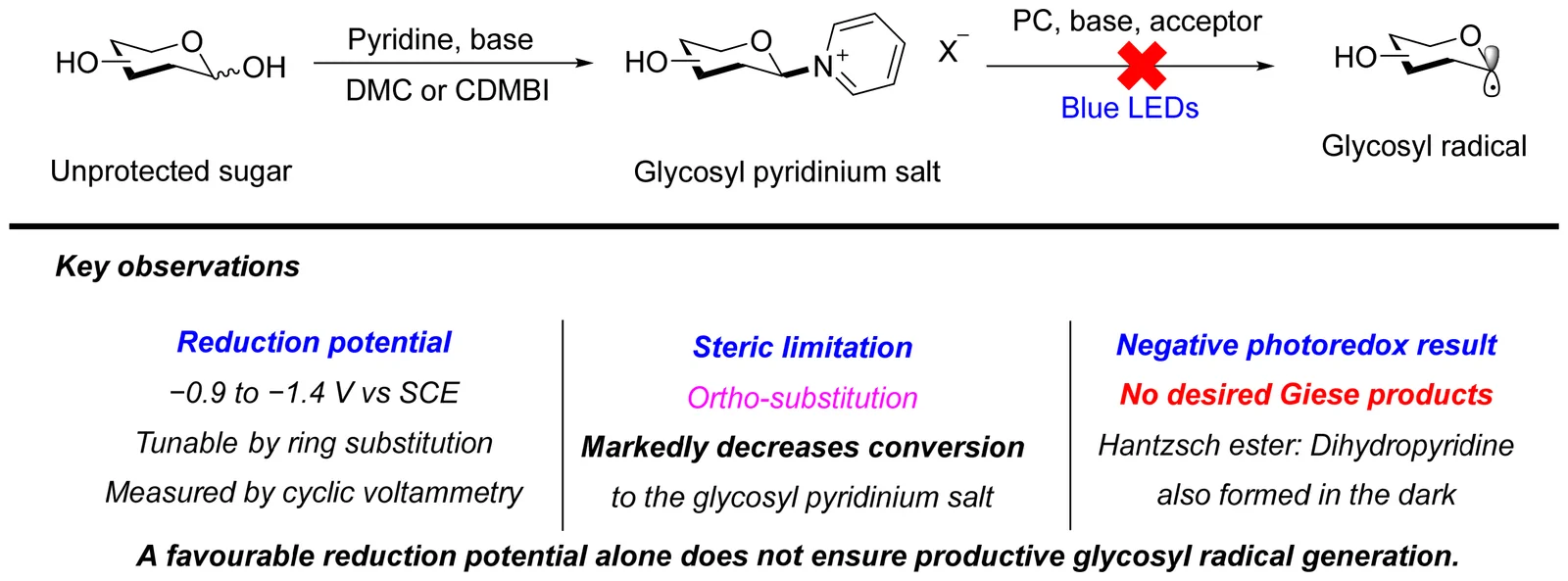
Preparation and testing of glycosyl pyridinium salts as radical precursors [51]. DMC/base allows salt formation, but the tested photoredox reactions did not give the intended Giese products. The reported Ered range is −1.40 to −0.93 V versus SCE for irreversible reductions. The rounded values in the diagram describe these irreversible reductions. “No desired Giese products” refers to the reaction outcome, not direct detection of a radical. CDMBI, 2-chloro-1,3-dimethylbenzimidazolinium chloride; PC, photocatalyst.

**Table 1:** Scope and main emphasis of related reviews.

Review (Year, Journal)	Primary Question Addressed	Comparison Basis	Coverage of One-Step Donor Generation from Native Sugars (2024–2026)
Koh et al. (2023, *Angew. Chem. Int. Ed.*) [21]	How can glycosyl radicals be diversified into different products?	Published scope	Predates period
Niu et al. (2023, *Acc. Chem. Res.*) [20]	Which bench-stable glycosyl radical donors are available?	Published scope	Predates period
Mandal et al. (2024, *Adv. Synth. Catal.*) [33]	What can sulfur-based bench-stable donors do?	Published scope	Sulfur donors; preparation burden discussed qualitatively
Du et al. (2025, *Asian J. Org. Chem.*) [34]	Which radical precursors enable glycosidation?	Published scope	Radical precursors; complementary donor-focused organization
Mukherjee et al. (2025, *Carbohydr. Res.*) [35]	What can glycosyl halides do under visible light?	Published scope	Halides only
Loh (2025, *Angew. Chem. Int. Ed.*) [37]	How can catalysts control glycosylation stereochemistry?	Published scope	Mainly protected donors
Fischer and Pedersen (2025, *Chem. Rev.*) [38]	What glycosylation is possible in water?	Published scope	Comprehensive for ionic aqueous chemistry; radical donors peripheral
Dey and Jayaraman (2025, *Synlett*) [39]	Which protecting-group-free reactions occur at the anomeric carbon?	Published scope	Ionic and base-mediated emphasis; radical C-glycosylation in one section
Mondal et al. (2025, *ChemistrySelect*) [40]	What can unprotected sugars be chemically transformed into?	Published scope	Glycosylation is one of twelve topics; no 2026 literature
Kärkäs et al. (2025, *Angew. Chem. Int. Ed.*) [41]	How can non-anomeric C–H bonds of sugars be functionalized site-selectively?	Published scope	Non-anomeric positions
Wang, Zhu and Yang (2026, *Chin. J. Org. Chem.*) [36]	Which unprotected radical donor classes enable protecting-group-free glycosylation?	Published scope	Fully unprotected radical donors; closely related scope [36]
This account	How do donor preparation, protection, activation, selectivity and isolation affect the full route from native sugar to product?	Primary studies through 31 August 2026	Primary focus, including 2026 primary literature
Li et al. (2026, *Org. Biomol. Chem.*) [43]	How do visible-light activation mechanisms vary across glycosyl radical precursors?	Post-cutoff contextual review	Activation-based comparison; additionally links native-sugar preparation, protection state and non-photochemical modes

The table compares subject matter and organizing emphasis. Publication year is not treated as evidence of a precisely known literature-search cutoff for another review.

**Table 4:** Representative anomeric linkage scope and the protection-state limits of the comparison.

Donor/Coupling State	C-Aryl	C-Alkyl	C-Alkenyl	S	Se	N	O	Refs
Sulfonyl hydrazide/UP	Yes	Yes	Yes	Yes	Yes	Not identified	Yes [25,52]	[27,28,29,30]
Sulfonyl hydrazide/MP then desilylation	Yes; α or β	Not tabulated	Not tabulated	Not tabulated	Not tabulated	Not tabulated	Not tabulated	[31]
Aryl thioglycoside/UP examples	Heteroaryl	Yes	Not identified	Not identified	Not identified	Not identified	Not identified	[19,54,55,56]
Sulfinate/protection depends on protocol	Yes	Yes	Not identified	Yes	Not identified	See §5.4; protection-specific	Not identified	[44,62,63,113]
DHP ester/UP	Not identified	Yes	Not identified	Not identified	Not identified	Not identified	Not identified	[45,70]
Transient Ti activation/native sugar	Not identified	Yes	Not identified	Not identified	Not identified	Not identified	Not identified	[79]
Phosphorus-assisted activation/protocol-dependent	Yes	Yes	Not identified	Not identified	Not identified	Yes; scope-specific	Not identified	[80,81,86]

A positive entry refers only to the cited demonstration, not to all donors or substrates in that architecture. “Not identified” means not established within the selected literature, not a demonstrated impossibility. The minimally protected Wang protocol [31] is a separate row and is not counted as fully unprotected coupling. Non-anomeric transformations are excluded. This table summarizes examples rather than an exhaustive linkage inventory.

**Table 5:** Selected sulfonyl hydrazide routes. Panel (**A**) compares preparation, protection and isolation; Panel (**B**) gives specific coupling conditions and outcomes. Compound and ligand numbers refer to the cited primary studies.

(**A**)			
**Study**	**Sequence and Protection**	**Isolation**	**Reported Preparation and Coupling**
Lu [30]	Glucose → donor 3a → empagliflozin 5as; 2 transformations; free OH groups throughout	Donor: EtOH recrystallization; product: silica, DCM/MeOH	Donor: 100 mmol glucose → 27.3 g, 74%; thermal coupling: 3.0 mmol ArBr → 920 mg, 68%
Guo [29]	Glucose → donor 25 → dapagliflozin 1; 2 transformations; free OH groups throughout	Donor: Precipitation/filtration; preparative product: silica; general scope: C18 chromatography	Donor: 100 mmol glucose → 33.1 g, 95%; coupling: 28.7 mmol donor → 7.2 g, 62%
Wang [31]	Hydrazidation → 4,6-O-silylation → coupling → desilylation; 4 transformations	Glucose and mannose sequences demonstrated with in situ intermediates and one final purification	Native-sugar sequences: glucose 3a, 45%; mannose 3c, 50%; model NMR yields: Panel B
Hong [28]	Sugar → donor → C-, S- or Se-glycoside; 2 transformations for sugar-derived sequence	Donor: trituration or silica, depending on substrate; coupling: silica, DCM/MeOH	Preparative examples: alkyl C-glycoside 22, 1.77 g (81%); Se-glycoside 29, 1.32 g (73%)
(**B**) Conditions and outcomes for the specified examples.			
**Example**	**Coupling Conditions**	**Reported Outcome**	**Basis and Source**
Lu [30], thermal 5as	20 mol% Ni(BF_4_)_2_·6H_2_O and L21; TEMP-OH 3 equiv.; donor 1.8 equiv.; DMF 0.2 M; 40 °C, 24 h	68% isolated; β:α > 20:1; 3.0 mmol ArBr basis	SI pp. 150–151; 68% based on ArBr, not excess sugar donor
Lu [30], light-assisted 5as	As above plus Ir photocatalyst 1.5 mol%; 445 nm LEDs; approximately 45 °C, 24 h	73% isolated; β:α > 20:1; 3.0 mmol ArBr basis	Same product and scale as thermal entry; separate photochemical conditions
Guo [29], preparative 1	20 mol% Ni(dme)Cl_2_ and L1; TMG 3 equiv.; ArI 1.5 equiv.; DMSO 0.1 M; 70 °C, overnight	62% isolated; 28.7 mmol donor. Optimization: 88%, β:α > 19:1	SI pp. 226–229; main Figure 2; optimization ratio not assigned to scale-up experiment
Wang [31], model 3a/4a	α: Ni complex A 20 mol%, PMP 5 equiv., MeCN; β: Ni/L3 20 mol%, TMG 3 equiv., DMF. 60 °C, 24 h; then Et3N·HF	α: 66% NMR, α:β 7:1; β: 60% NMR, β:α 10:1	Entries 3 and 6; major-anomer NMR yields after coupling/desilylation; not total isolated route yields
Hong [28], alkenylation	CuCl and L1, 1 equiv. each; KOH 3 equiv.; alkyne 2 equiv.; HFIP 0.1 M; 90 °C, 24 h	0.1 mmol model: 88% isolated, E:Z > 20:1; preparative example: 64%, α, E:Z 20:1	SI pp. 9–10, 27; alkene geometry and anomeric configuration are separate descriptors
Hong [28], alkylation	Cu(OTf)_2_ 0.5 equiv.; NaOAc 3 equiv.; alkene 2 equiv.; THF or MeCN/H_2_O (2:1), 0.1 M; 80 °C, 12 h	0.1 mmol model: 83% isolated; product 22: 1.77 g, 81%, α	SI pp. 11–12, 27; product-specific scale data; no blanket yield assigned to C/S/Se scope

Yields are isolated unless marked as NMR. Catalyst and reagent quantities refer to the limiting substrate defined in each procedure. L1, L3 and L21 are the ligand labels used in the respective publications and do not denote the same structures across studies. The specific structures should be verified against the original references. TEMP-OH is 2,2,6,6-tetramethylpiperidin-4-ol; PMP is 1,2,2,6,6-pentamethylpiperidine; TMG is tetramethylguanidine.

## Data Availability

No new data were created or analyzed in this study. Data sharing is not applicable to this article.
